# Genetic characterization of influenza A virus subtypes H11N6, H11N7, and H11N9 isolated from free‐grazing ducks, Thailand

**DOI:** 10.1111/irv.12960

**Published:** 2022-01-10

**Authors:** Supassama Chaiyawong, Kamonpan Charoenkul, Kitikhun Udom, Ekkapat Chamsai, Waleemas Jairak, Supanat Boonyapisitsopa, Napawan Bunpapong, Alongkorn Amonsin

**Affiliations:** ^1^ Department of Veterinary Public Health, Faculty of Veterinary Science Chulalongkorn University Bangkok Thailand; ^2^ Emerging and Re‐emerging Infectious Diseases in Animals, Center of Excellence, and One Health Research Cluster, Faculty of Veterinary Science Chulalongkorn University Bangkok Thailand; ^3^ Veterinary Diagnostic Laboratory, Faculty of Veterinary Science Chulalongkorn University Bangkok Thailand

**Keywords:** characterization, ducks, H11N6, H11N7, H11N9, influenza

## Abstract

Influenza A viruses (IAVs) infect avian species and several mammalian species including humans. Anseriformes water birds are an important reservoir of IAVs. In this study, we identified and characterized IAV subtypes H11N6 (n = 5), H11N7 (n = 3), and H11N9 (n = 3) isolated during the influenza surveillance program in free‐grazing ducks from 2012 to 2015 in Thailand. Eleven IAV‐H11 viruses were characterized by either whole genome sequencing (n = 5) or HA and NA gene sequencing (n = 6) for phylogenetic and amino acid analyses. Phylogenetic analysis showed that Thai IAV‐H11 were grouped into Avian Eurasian lineage. Amino acid analysis showed that all Thai IAV‐H11 viruses have low pathogenic avian influenza (LPAI) characteristics and sensitive to Oseltamivir and Amantadine. Novel reassortant viruses (IAV‐H11N7 and IAV‐H11N9) have been observed. The reassortant viruses contained NP, M, and NS gene segments which originate from intercontinental sources which never been reported in Thai IAVs. In summary, this study demonstrated high genetic diversity of IAV‐H11 circulating in free‐grazing ducks. Free‐grazing ducks infected with IAVs generated novel reassortant IAV‐H11. Thus, surveillance of IAVs in free‐grazing ducks should be routinely conducted to monitor novel reassortant viruses and subsequently potential virulence viruses.

## 1 INTRODUCTION

Influenza A virus (IAV) or Alphainfluenzavirus is an enveloped, segmented, single‐stranded RNA virus of the family *Orthomyxoviridae*. The virus can infect avian species and several mammal species including humans. IAVs can be classified into subtypes based on hemagglutinin and neuraminidase proteins (18HA and 11NA).^1^, ^2^, ^3^, ^4^ Several IAV subtypes, H1–H16 and N1–N9, circulate in avian species especially in order *Anseriformes*. Waterfowls are susceptible to infection by IAVs and serve as natural reservoirs and can spread the viruses to other avian and mammal species. It has been documented that IAV reservoir hosts are important for generating novel reassortant viruses.^5^, ^6^, ^7^

IAV subtype H11 (IAV‐H11) have been reported in avian species worldwide. Most of the IAV‐H11 were found in avian species of order *Anseriformes* and a few were reported in order *Charadriiformes*, and rarely were found in order *Galliformes*.^8^ It has also been reported that IAV‐H11 rarely infect humans. For example, IAV‐H11 had been reported to infect humans who were exposed to wild birds.^8^ The previous study showed that receptor binding site of IAV‐H11 has preferential binding to both avian (SA α 2,3‐Gal) and mammalian (SA α 2,6‐Gal) receptors.^9^ In addition, IAV‐H11N6 had been reported to infect pigs in South Korea.^10^ The IAV‐H11 viruses (H11N2, H11N3, and H11N9) have been reported in domestic ducks from live bird markets in China.^11^, ^12^, ^13^

In Thailand, IAV‐H11 were isolated from free‐grazing ducks (FGDs) during the influenza surveillance program in 2012–2015. The FGDs (mainly *Anas platyrhynchos*) are semidomestic ducks raised in open rice field in flocks of >1000 birds for egg and meat production in Southeast Asia. The ducks are raised to free graze for food in rice fields after harvesting and frequently moved among rice fields for new food sources. FGDs potentially interface with wild birds and domestic birds such as backyard ducks, chickens, and quails. Because FGDs are reservoirs of the IAVs, therefore, interspecies transmission of IAVs commonly occurred.^14^, ^15^, ^16^, ^17^, ^18^, ^19^ It is also noted that FGDs could be infected with HPAI‐H5N1 without clinical signs and can carry virulent viruses to other animals. Thus, novel reassortant viruses with virulence genes can arise.^20^, ^21^ In this study, we selected IAV‐H11 (H11N6, H11N7, and H11N9) isolated from FGDs in Thailand and performed genetic characterization of IAV‐H11 by whole genome sequencing to determine genetic diversity of the viruses and subsequently monitor potential virulence viruses.

## 2 MATERIAL AND METHODS

### 2.1 Viruses

The IAVs were isolated from oropharyngeal swabs (OP) and cloacal swabs (CS) collected from FGDs during 2012–2015 influenza surveillance program in Thailand. The FGD flocks were selected based on owner collaboration, and approximately 50 ducks from each flock were randomly selected and sampled. The IAVs were isolated by egg inoculation into 9‐ to 11‐day‐old embryonated chicken eggs.^22^ The allantoic fluid was tested for influenza virus by hemagglutination (HA) test. The allantoic fluid with the HA titer ≥2 HA unit were interpreted as positive influenza virus. The HA positive samples were confirmed for the presence of IAV by using real‐time Reverse Transcriptase ‐PCR (rRT‐PCR) specific to the Matrix (M) gene.^23^ In this study, IAVs (n = 11) isolated from FGDs were included for genetic characterization. This study was conducted under the approval of the Chulalongkorn University Animal Care and Use Protocol (IACUC# 2031050 and 2031051).

### 2.2 IAV detection

RNA extraction from the allantoic fluid was carried out by Nucleospin® RNA virus (Macherey‐Nagel, Germany) according to the manufacturer's instruction. The viral RNA was subjected to IAV detection by rRT‐PCR specific to Matrix (M) gene.^23^ In brief, the rRT‐PCR was performed by using the SuperScript® III Platinum® One‐Step Quantitative RT‐PCR System (Invitrogen®). The 30 μl of reagent mixture contained 4 μl of RNA template, 1× master mix buffer, 0.8 μM of M gene specific primers, 0.2 μM of probe, 0.6 mM MgSO_4_, 1 unit of Superscript III reverse transcriptase, and RNase‐free water. Amplification was done by rRT‐PCR which contained three steps: (1) reverse transcription at 50°C for 30 min, (2) predenaturation at 95°C for 15 min, and (3) denaturation for 50 cycles of 95°C for 15 s and annealing–extension at 60°C for 30 s. The rRT‐PCR results were evaluated by cycle threshold (Ct), and values less than 36 were considered positive, greater than 40 were negative, and between 36 and 40 were interpreted as inconclusive.

### 2.3 IAV subtype identification

The positive RNA samples were synthesized for cDNA by using Improm‐II Reverse Transcription System (Promega, Madison, WI, USA) with universal primer for IAVs. The cDNA samples were subjected to IAV subtype identification. The specific primers of each influenza subtypes, H1–H15 and N1–N9, were used for influenza subtyping by PCR using primers previously described.^24^, ^25^, ^26^ In detail, 30 μl of PCR mixture contained 1 μl of cDNA, 1× master mix buffer (TopTaq ™), 0.8 μM of primers for each subtype, and distilled water. The PCR conditions were 94°C for 3 min and 35 cycles of 94°C for 30 s, 50°C (for H1–H15) or 45°C (for N1–N9) for 30 s, and 72°C for 30 s. The PCR product was run in 1.2% of agarose gel with Red safe in 0.5× Tris borate EDTA (TBE).

### 2.4 IAV characterization

Eight segments of the IAVs were amplified using TopTaq master mix (Qiagen, Hilden, Germany) with specific primer sets and newly designed primers for sequencing.^27^ In brief, 30 μl of PCR mixture contained 1.5 μl of cDNA, 1.2 μM of each forward and reverse primers, 1× Top Taq Master Mix (QIAGEN), 1× loading dye, and distilled water. PCR condition was set as initial denaturation at 94°C for 3 min; 35 cycles of denaturation at 94°C for 30 s, annealing temperature depend on primers for 45 s, and extension at 72°C for 1 min; and final extension at 72°C for 7 min. PCR products were then visualized by gel electrophoresis with 1.2% of agarose gel in 0.5× Tris borate EDTA. The amplicons were purified using Nucleospin® PCR clean up kit. The purified PCR products were then sequenced using BigDye® Terminator v3.1 cycle sequencing kit (1st Base Laboratories, Kembangan, Malaysia). The nucleotide sequences of each gene segment were validated and assembled using SeqMan software v.5.03 (DNASTAR Inc., Madison, WI, USA).

To determine nucleotide identity, the nucleotide sequences of each gene segment were compared with those of the reference IAVs from the GenBank database by using nucleotide BLAST tool. For pairwise comparison, nucleotide sequences and deduced amino acids of the IAVs in this study were aligned with those of reference IAV sequences from different subtypes and geographic locations (Eurasian and North America lineages) by using Muscle version 3.6 and MegAlign version 5.03 (DNASTARInc., Madison, WI, USA) software. Phylogenetic trees of eight gene segments were generated by using MEGA v10.0 applying neighbor‐joining method with 1000 replications for bootstrap analysis. The reference data set for phylogenetic analysis was selected to represent geographic locations (Eurasian and North America lineages) and available sequences of Thai IAV‐H11Nx, HxN6, HxN7, and HxN9.

## 3 RESULTS

**FIGURE 1:**
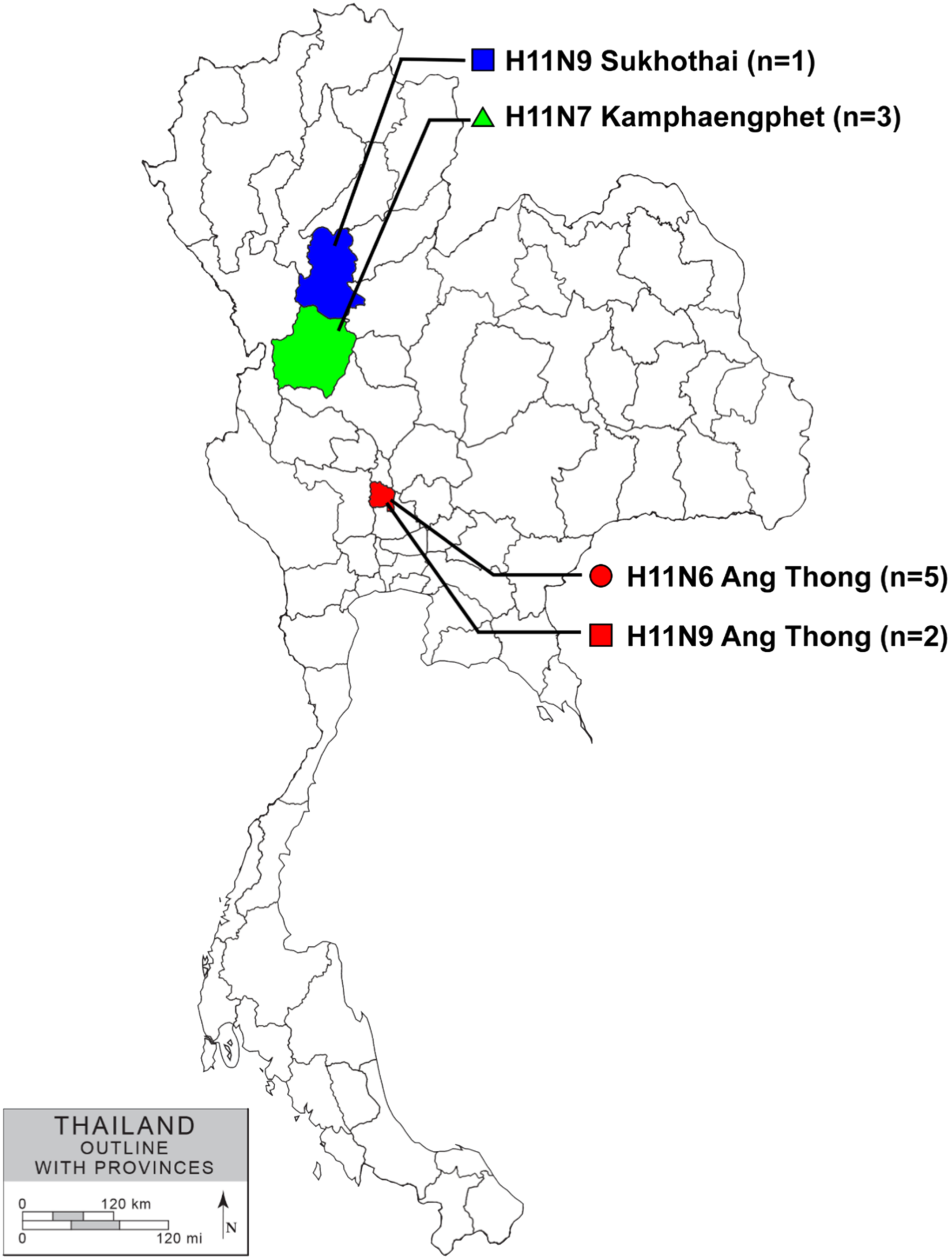
Map of Thailand and provinces where the Thai IAV‐H11 were isolated in this study

In this study, we tested oropharyngeal swabs (n = 780) and cloacal swabs (n = 780) collected from 18 FGD flocks during 2012–2015 (Table S1). We detected and isolated IAVs (n = 11) from FGD flocks sampled from three provinces of central and northern Thailand (Ang Thong, Sukhothai, and Kamphaengphet) (Table 1 and Figure 1). All viral RNA samples were confirmed as IAV by rRT‐PCR assay and had cycle threshold value ≤20. Then, the IAVs were subtyped as IAV‐H11N6 (n = 5), IAV‐H11N7 (n = 3), and IAV‐H11N9 (n = 3). The representative viruses IAV‐H11N6 (n = 2), IAV‐H11N7 (n = 1), and IAV‐H11N9 (n = 2) were selected for whole genome sequencing, and the other IAV‐H11 (n = 6) were subjected to HA and NA gene sequencing. The nucleotide sequences of IAV‐H11 were submitted to the GenBank database under the accession# MW857483‐857534 (Table 1).

**TABLE 1:** Description of Thai IAV‐H11 from free‐grazing ducks characterized in this study

Virus	Strain name	Subtype	Year	Location	Flock	Source	Date	Sequencing	GenBank #
CU‐12657 (H11N6)	A/duck/Thailand/CU‐12657T/2012	H11N6	2012	Ang Thong	1	OP	Oct 2012	WGS	MW857483‐90
CU‐12658 (H11N6)	A/duck/Thailand/CU‐12658T/2012	H11N6	2012	Ang Thong	1	OP	Oct 2012	HA, NA	MW857491‐92
CU‐12661 (H11N6)	A/duck/Thailand/CU‐12661T/2012	H11N6	2012	Ang Thong	1	OP	Oct 2012	HA, NA	MW857493‐94
CU‐12677 (H11N6)	A/duck/Thailand/CU‐12677T/2012	H11N6	2012	Ang Thong	2	OP	Oct 2012	WGS	MW857495‐02
CU‐12678 (H11N6)	A/duck/Thailand/CU‐12678C/2012	H11N6	2012	Ang Thong	2	CS	Oct 2012	HA, NA	MW857503‐04
CU‐12660 (H11N9)	A/duck/Thailand/CU‐12660T/2012	H11N9	2012	Ang Thong	1	OP	Oct 2012	WGS	MW857505‐12
CU‐12662 (H11N9)	A/duck/Thailand/CU‐12662T/2012	H11N9	2012	Ang Thong	1	OP	Oct 2012	HA, NA	MW857513‐14
CU‐14442 (H11N9)	A/duck/Thailand/CU‐14442C/2014	H11N9	2014	Sukhothai	3	CS	Feb 2014	WGS	MW857515‐22
CU‐16340 (H11N7)	A/duck/Thailand/CU‐16340C/2015	H11N7	2015	Kamphaengphet	4	CS	Feb 2015	WGS	MW857523‐30
CU‐16345 (H11N7)	A/duck/Thailand/CU‐16345C/2015	H11N7	2015	Kamphaengphet	4	CS	Feb 2015	HA, NA	MW857531‐32
CU‐16347 (H11N7)	A/duck/Thailand/CU‐16347C/2015	H11N7	2015	Kamphaengphet	4	CS	Feb 2015	HA, NA	MW857533‐34

Abbreviations: CS, cloacal swabs; HA, hemagglutination; NA, neuraminidase; OP, oropharyngeal swabs; WGS, characterization by whole genome sequencing.

### 3.1 Genetic characteristics of HA gene of Thai IAV‐H11

**FIGURE 2:**
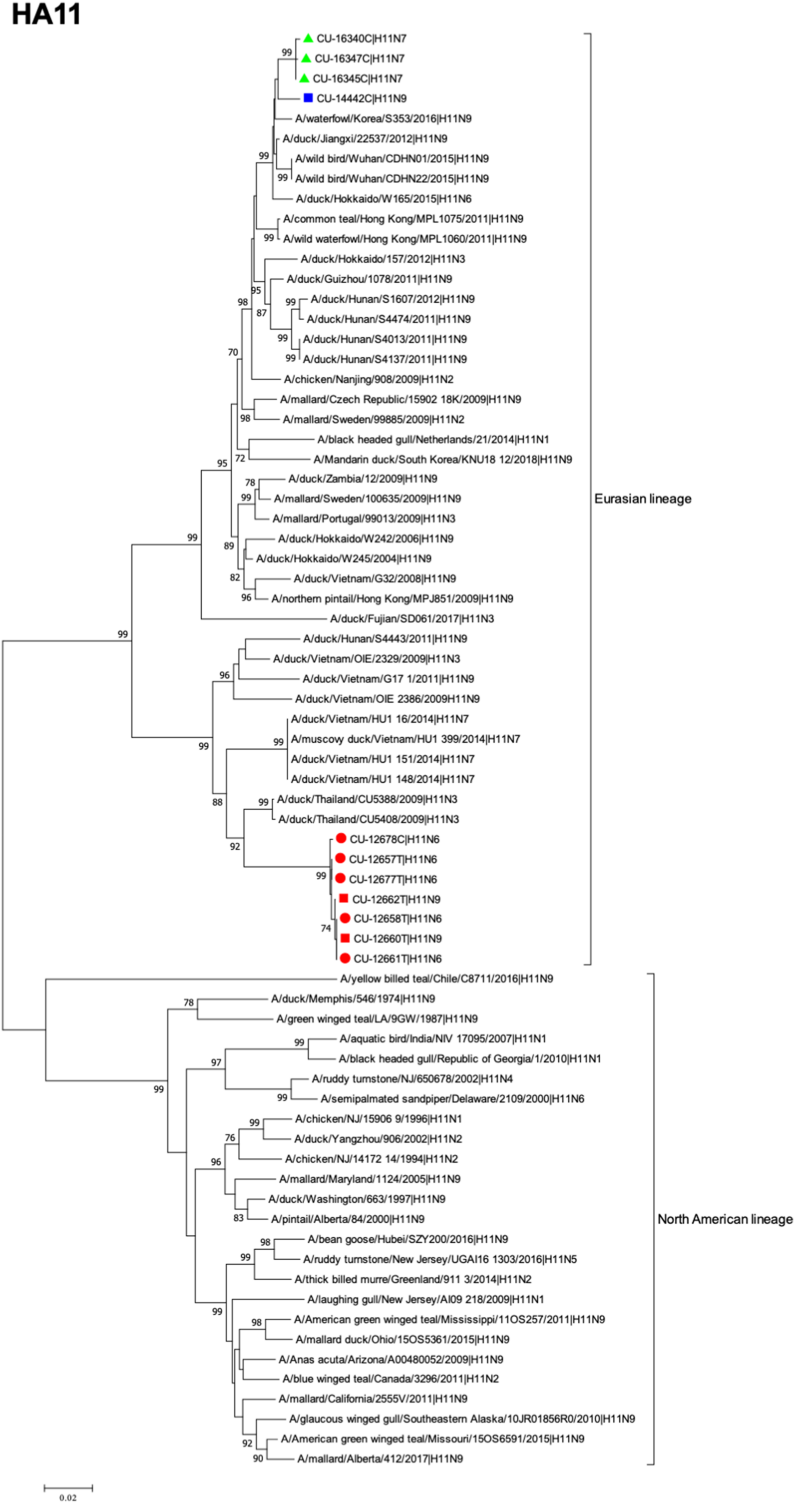
Phylogenetic tree of H11 gene of Thai IAV‐H11. The phylogenetic tree was generated by the neighbor‐joining algorithm with 1000 replications of bootstrap analysis by using the MEGA7.0 program. Red circle represents IAV‐H11N6, red square represents IAV‐H11N9 (subgroup 1), blue square represents IAV‐H11N9 (subgroup 2), and green triangles represent IAV‐H11N7

For H11 gene, Thai IAV‐H11 viruses possessed high nucleotide identities to IAVs of Eurasian lineage (Tables S2 and S3). Phylogenetic tree of the H11 gene showed that the viruses can be divided into North American lineage and Eurasian lineage. The Thai IAV‐H11 were clustered with the IAVs of the Eurasian lineage. It is noted that Thai IAV‐H11 could be further grouped into two different subgroups. The subgroup 1 (Ang Thong subgroup) included IAV‐H11N6 (n = 5) and IAV‐H11N9 (n = 2), which were recovered from the same province (Ang Thong). These viruses were closely related to the Thai IAV‐H11N3 isolated in 2009 (95.44–95.68%). The subgroup 2 (Sukhothai and Kamphaeng Phet subgroup) included IAV‐H11N9 (n = 1) and H11N7 (n = 3), which were closely related to the Chinese H11N9 isolated in 2012 (98.78–98.88%) (Figure 2).

### 3.2 Genetic characteristics of NA gene of Thai IAV‐H11

**FIGURE 3:**
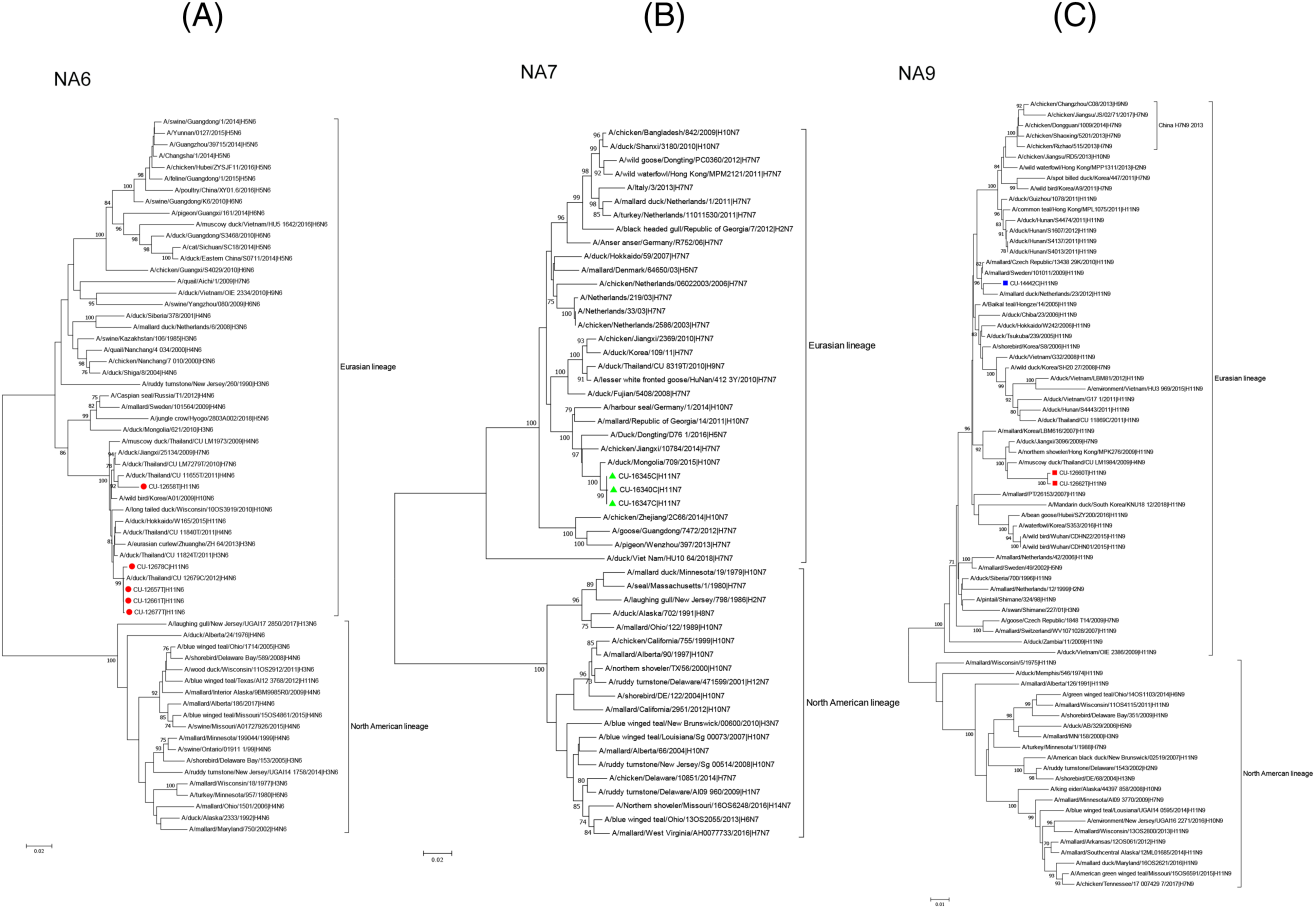
(A) Phylogenetic tree of N6 gene of Thai IAV‐H11. (B) Phylogenetic tree of N7 gene of Thai IAV‐H11. (C) Phylogenetic tree of N9 gene of Thai IAV‐H11. The phylogenetic tree was generated by the neighbor‐joining algorithm with 1000 replications of bootstrap analysis by using the MEGA7.0 program. Red circle represents IAV‐H11N6, red square represents IAV‐H11N9 (subgroup 1), blue square represents IAV‐H11N9 (subgroup 2), and green triangles represent IAV‐H11N7

The phylogenetic tree of N6 gene can be divided into North American lineage and Eurasian lineage. The N6 gene of Thai IAV‐H11N6 (n = 5) were clustered in avian Eurasian lineage and were closely related to Thai IAV‐H4N6. For N7 gene, the phylogenetic tree of N7 gene possessed two major groups, the North American and Eurasian lineages. The Thai IAV‐H11N7 (n = 3) were clustered in avian Eurasian lineage and were closely related with IAV‐H10N7 from Mongolia. For N9 gene, the phylogenetic tree of N9 gene can be divided into North American lineage and Eurasian lineage. The Thai IAV‐H11N9 (n = 3) were clustered in avian Eurasian lineage. It is noted that the Thai IAV‐H11N9 could be grouped into two subgroups: subgroup 1 (IAV‐H11N9 from Ang Thong) and subgroup 2 (IAV‐H11N9 from Sukhothai). Both subgroups were in a separated group to IAV‐ H7N9 in China (Figure 3).

### 3.3 Genetic characteristics of internal gene segments of Thai IAV‐H11

**FIGURE 4:**
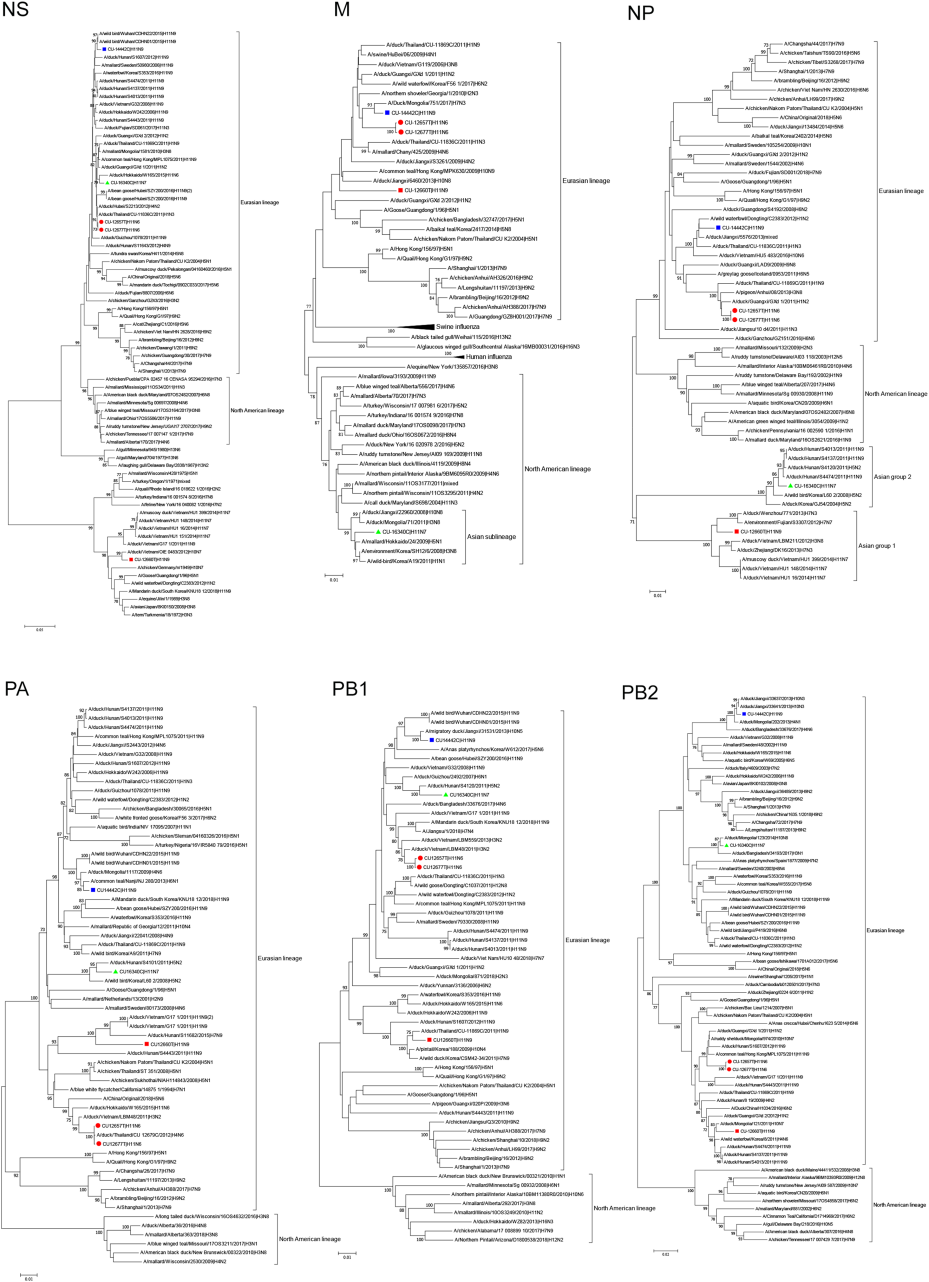
Phylogenetic tree of internal gene segments of Thai IAV‐H11. The phylogenetic tree was generated by the neighbor‐joining algorithm with 1000 replications of bootstrap analysis by using the MEGA7.0 program. Red circle represents IAV‐H11N6, red square represents IAV‐H11N9 (subgroup 1), blue square represents IAV‐H11N9 (subgroup 2), and green triangles represent IAV‐H11N7

Phylogenetic trees of internal gene segments of IAV‐H11 revealed possible reassortment of Thai IAV‐H11 (Table 2). The common origin of each gene lineages of Thai IAVs is Eurasian lineage (EA). However, in this study, some internal gene segments of IAV‐H11N9 (CU‐12660) and IAV‐H11N7 (CU‐16340) were originated from uncommon Eurasian and North American lineages. Phylogenetically, the NS gene of IAV‐H11N9 (CU‐12660) belongs to the avian Eurasian lineage (allele B) (EA‐B) but not common avian Eurasian lineage (allele A) (EA) (CU‐12657, CU‐12677, CU‐14442, and CU‐16340). The M gene of IAV‐H11N7 (CU16340) belongs to the avian North American lineage (NA) which closely related to A/mallard/Hokkaido/24/2009 (H5N1). Phylogenetic tree of NP gene can be divided into four lineages: (1) North American lineage (NA), (2) Eurasian lineage (EA), (3) Asian group 1 (A1), and (4) Asian group 2 (A2). The NP genes of IAV‐H11N9 (CU‐12660) and H11N7 (CU‐16340) belong to Asian group 1 (A1) and Asian group 2 (A2), respectively (Figure 4).

**TABLE 2:** Genetic constellation of Thai IAV‐H11 representing the lineages of all eight gene segments

Virus	Subtype	Year	Location	PB2	PB1	PA	HA	NP	NA	M	NS
CU‐12657 (H11N6)	H11N6	2012	Ang Thong	EA	EA	EA	EA	EA	EA	EA	EA
CU‐12677 (H11N6)	H11N6	2012	Ang Thong	EA	EA	EA	EA	EA	EA	EA	EA
CU‐12660 (H11N9)	H11N9	2012	Ang Thong	EA	EA	EA	EA	A1	EA	EA	EA‐B
CU‐14442 (H11N9)	H11N9	2014	Sukhothai	EA	EA	EA	EA	EA	EA	EA	EA
CU‐16340 (H11N7)	H11N7	2015	Kamphaengphet	EA	EA	EA	EA	A2	EA	NA	EA
CU‐12658 (H11N6)	H11N6	2012	Ang Thong				EA		EA		
CU‐12661 (H11N6)	H11N6	2012	Ang Thong				EA		EA		
CU‐12678 (H11N6)	H11N6	2012	Ang Thong				EA		EA		
CU‐12658 (H11N6)	H11N6	2012	Ang Thong				EA		EA		
CU‐12662 (H11N9)	H11N9	2012	Ang Thong				EA		EA		
CU‐16345 (H11N7)	H11N7	2015	Kamphaengphet				EA		EA		
CU‐16347 (H11N7)	H11N7	2015	Kamphaengphet				EA		EA		

Abbreviations: A1, avian group 1; A2, avian group 2; EA, avian Eurasian lineage (allele A); EA‐B; avian Eurasian lineage (allele B); NA, avian North American lineage.

### 3.4 Amino acid analysis of Thai IAV‐H11

In this study, amino acid analysis for the translated genetic sequences of the Thai IAV‐H11 was conducted. At the HA cleavage site, the IAV‐H11 possessed “PAIASR/GLF” suggesting low pathogenic avian influenza (LPAI) characteristic. Analysis of receptor binding site showed that Thai IAV‐H11 contained Q226 and G228 suggesting preferential binding for α 2‐3‐linked sialic acid receptor or avian receptor.^28^, ^29^ Moreover, Thai IAV‐H11N7 had single amino acid difference near the receptor binding site (134–138; GVTAS) which is different from that of other IAV‐H11 (134–138; GVTAA) (Table 3). For NA gene analysis, the Thai IAV‐H11 did not contain amino acid deletion in the NA stalk region and amino acid substitutions associated with neuraminidase resistance suggesting that the IAV‐H11 were sensitive to Oseltamivir.^30^, ^31^ For PB2 gene analysis, the PB2‐627 of IAV‐H11 contained glutamic acid (E), whereas one IAV‐H11N9 (CU‐12660) contained glycine (G).^32^, ^33^ Interestingly, glycine at PB2‐627 position is very rare because most amino acid at PB2‐627 is 627E in avian viruses and 627K in mammalian viruses (Table 3).

**TABLE 3:** Amino acid analysis of Thai IAV‐H11 at the HA, NA, and internal gene segments

Virus	Subtype	Location	Host	Year	HA cleavage site	Receptor‐binding site (RBS)							Left edge of RBS	Right edge of RBS
					320–329	98	153	155	183	190	194	195	224–229	134–138
North American consensus	H11Nx		Avian	—	PAIATR	Y	W	I	H	E	L	Y	NGQAGR	GVTAA
Eurasian consensus	H11Nx		Avian	—	PAIASR	Y	W	I	H	E	L	Y	NGQAGR	GVTAA
Hokkaido/W165/15	H11N6	Japan	Duck	2015	PAIASR	Y	W	I	H	E	L	Y	NGQAGR	GVTAA
Vietnam/HU1‐399/14	H11N7	Vietnam	Duck	2014	PAIASR	Y	W	I	H	E	L	Y	NGQAGR	GVTAA
Vietnam/HU1‐151/14	H11N7	Vietnam	Duck	2014	PAIASR	Y	W	I	H	E	L	Y	NGQAGR	GVTAA
Hongkong/MPL1075/11	H11N9	Hong Kong	Teal	2011	PAIASR	Y	W	I	H	E	L	Y	NGQAGR	GVTAA
Hunan/S4443/11	H11N9	China	Duck	2011	PAIASR	Y	W	I	H	E	L	Y	NGQAGR	GVTAA
Jiangxi/22537/12	H11N9	China	Duck	2012	PAIASR	Y	W	I	H	E	L	Y	NGQAGR	GVTAA
Korea/KNU18‐12/18	H11N9	Korea	Duck	2018	PAIASR	Y	W	I	H	E	L	Y	NGQAGR	GVTAA
Rizhao/515/13	H7N9	China	Chicken	2013	PEIPKGR	Y	W	L	H	E	L	Y	NGLSGR	GVTSA
Jiangsu/JS‐02/17	H7N9	China	Chicken	2017	PEIPKGR	Y	W	L	H	E	L	Y	NGLSGR	GVTGA
This study														
CU‐12657 (H11N6)	H11N6	Ang Thong	Duck	2012	PAIASR	Y	W	I	H	E	L	Y	NGQAGR	GVTAA
CU‐12658 (H11N6)	H11N6	Ang Thong	Duck	2012	PAIASR	Y	W	I	H	E	L	Y	NGQAGR	GVTAA
CU‐12661 (H11N6)	H11N6	Ang Thong	Duck	2012	PAIASR	Y	W	I	H	E	L	Y	NGQAGR	GVTAA
CU‐12677 (H11N6)	H11N6	Ang Thong	Duck	2012	PAIASR	Y	W	I	H	E	L	Y	NGQAGR	GVTAA
CU‐12678 (H11N6)	H11N6	Ang Thong	Duck	2012	PAIASR	Y	W	I	H	E	L	Y	NGQAGR	GVTAA
CU‐12660 (H11N9)	H11N9	Ang Thong	Duck	2012	PAIASR	Y	W	I	H	E	L	Y	NGQAGR	GVTAA
CU‐12662 (H11N9)	H11N9	Ang Thong	Duck	2012	PAIASR	Y	W	I	H	E	L	Y	NGQAGR	GVTAA
CU‐14442 (H11N9)	H11N9	Sukhothai	Duck	2014	PAIASR	Y	W	I	H	E	L	Y	NGQAGR	GVTAS
CU‐16340 (H11N7)	H11N7	Kamphaengphet	Duck	2015	PAIASR	Y	W	I	H	E	L	Y	NGQAGR	GVTAS
CU‐16345 (H11N7)	H11N7	Kamphaengphet	Duck	2015	PAIASR	Y	W	I	H	E	L	Y	NGQAGR	GVTAS
CU‐16347 (H11N7)	H11N7	Kamphaengphet	Duck	2015	PAIASR	Y	W	I	H	E	L	Y	NGQAGR	GVTAS

Virus	Subtype	Location	Host	Year	Stalk region	Oseltamivir resistance^a^			
					49–68	119	222	274	292
North American consensus	H11Nx		Avian	—	No del	E	I	H	R
Eurasian consensus	H11Nx		Avian	—	No del	E	I	H	R
Hokkaido/W165/15	H11N6	Japan	Duck	2015	No del	E	I	H	R
Vietnam/HU1‐399/14	H11N7	Vietnam	Duck	2014	No del	E	I	H	R
Vietnam/HU1‐151/14	H11N7	Vietnam	Duck	2014	No del	E	I	H	R
Hongkong/MPL1075/11	H11N9	Hong Kong	Teal	2011	No del	E	I	H	R
Hunan/S4443/11	H11N9	China	Duck	2011	No del	E	I	H	R
Jiangxi/22537/12	H11N9	China	Duck	2012	No del	E	I	H	R
Korea/KNU18‐12/18	H11N9	Korea	Duck	2018	No del	E	I	H	R
Rizhao/515/13	H7N9	China	Chicken	2013	No del	E	I	H	R
Jiangsu/JS‐02/17	H7N9	China	Chicken	2017	No del	E	I	H	R
This study									
CU‐12657 (H11N6)	H11N6	Ang Thong	Duck	2012	No del	E	I	H	R
CU‐12658 (H11N6)	H11N6	Ang Thong	Duck	2012	No del	E	I	H	R
CU‐12661 (H11N6)	H11N6	Ang Thong	Duck	2012	No del	E	I	H	R
CU‐12677 (H11N6)	H11N6	Ang Thong	Duck	2012	No del	E	I	H	R
CU‐12678 (H11N6)	H11N6	Ang Thong	Duck	2012	No del	E	I	H	R
CU‐12660 (H11N9)	H11N9	Ang Thong	Duck	2012	No del	E	I	H	R
CU‐12662 (H11N9)	H11N9	Ang Thong	Duck	2012	No del	E	I	H	R
CU‐14442 (H11N9)	H11N9	Sukhothai	Duck	2014	No del	E	I	H	R
CU‐16340 (H11N7)	H11N7	Kamphaengphet	Duck	2015	No del	E	I	H	R
CU‐16345 (H11N7)	H11N7	Kamphaengphet	Duck	2015	No del	E	I	H	R
CU‐16347 (H11N7)	H11N7	Kamphaengphet	Duck	2015	No del	E	I	H	R

Virus	Subtype	Location	Host	Year	M gene					PB2		NS1	
					Amantadine resistance^b^					Virulence determinant		Virulence determinant	
					26	27	30	31	34	627	701	80–84	92
North American consensus	H11Nx		Avian	—	Q	R	D	V	G	E	D	No del	D
Eurasian consensus	H11Nx		Avian	—	Q	R	D	V	G	E	D	No del	D
Hokkaido/W165/15	H11N6	Japan	Duck	2015	Q	R	D	V	G	E	D	No del	D
Vietnam/HU1‐399/14	H11N7	Vietnam	Duck	2014	Q	R	D	V	G	E	NA	No del	D
Vietnam/HU1‐151/14	H11N7	Vietnam	Duck	2014	Q	R	D	V	G	E	D	No del	D
Hongkong/MPL1075/11	H11N9	Hong Kong	Teal	2011	Q	R	D	V	G	E	D	No del	D
Hunan/S4443/11	H11N9	China	Duck	2011	Q	R	D	V	G	E	D	No del	D
Jiangxi/22537/12	H11N9	China	Duck	2012	Q	R	D	V	G	E	D	No del	D
Korea/KNU18‐12/18	H11N9	Korea	Duck	2018	Q	R	D	V	G	E	D	No del	D
Rizhao/515/13	H7N9	China	Chicken	2013	Q	R	D	V	G	E	D	No del	D
Jiangsu/JS‐02/17	H7N9	China	Chicken	2017	Q	R	D	V	G	E	D	No del	D
This study													
CU‐12657 (H11N6)	H11N6	Ang Thong	Duck	2012	Q	R	D	V	G	E	D	No del	D
CU‐12658 (H11N6)	H11N6	Ang Thong	Duck	2012	—	—	—	—	—	—	—	—	—
CU‐12661 (H11N6)	H11N6	Ang Thong	Duck	2012	—	—	—	—	—	—	—	—	—
CU‐12677 (H11N6)	H11N6	Ang Thong	Duck	2012	Q	R	D	V	G	E	D	No del	D
CU‐12678 (H11N6)	H11N6	Ang Thong	Duck	2012	—	—	—	—	—	—	—	—	—
CU‐12660 (H11N9)	H11N9	Ang Thong	Duck	2012	Q	R	D	V	G	E	D	No del	D
CU‐12662 (H11N9)	H11N9	Ang Thong	Duck	2012	—	—	—	—	—	—	—	—	—
CU‐14442 (H11N9)	H11N9	Sukhothai	Duck	2014	Q	R	D	V	G	E	D	No del	D
CU‐16340 (H11N7)	H11N7	Kamphaengphet	Duck	2015	Q	R	D	V	G	E	D	No del	D
CU‐16345 (H11N7)	H11N7	Kamphaengphet	Duck	2015	—	—	—	—	—	—	—	—	—
CU‐16347 (H11N7)	H11N7	Kamphaengphet	Duck	2015	—	—	—	—	—	—	—	—	—

^a^ Oseltamivir resistant amino acids: E119V, I222L, H274Y, and R292K.

^b^ Amantadine resistant amino acids: Q26F, R27A, D30T/V, V31N, and G34E.

The analysis of genomic signatures related to host specificity of the internal gene segments of IAV‐H11 showed that the IAV‐H11N9 (CU14442) contained lysine (K) at position NP109, which is rare in avian viruses (Table 4). For IAV‐H11N7 (CU16340), the virus contained four unusual amino acids at NP293 (K), NP305 (H), NP313 (L), and NP455 (E), which NP293 (K) and NP455 (E) were predominantly observed in human viruses. It is noted that NP305 (H) and NP313 (L) were rarely reported in any IAVs in the GenBank (Table 3).

**TABLE 4:** Amino acid analysis of genomic signatures of avian IAV‐H11

			PB2									PB1				
Virus	Subtype	Location	44	199	271	475	567	588	613	627	702	327	336			
Avian IAVs			A	A	T	L	D	A	V	E	K	R	V			
Human IAVs			S	S	A	M	N	I	T	K	R	K	I			
pdmH1N1 2009	H1N1	Ang Thong	A	A	A	L	D	T	V	E	K	R	I			
CU‐12657 (H11N6)	H11N6	Ang Thong	A	A	T	L	D	A	V	E	K	R	V			
CU‐12677 (H11N6)	H11N6	Ang Thong	A	A	T	L	D	A	V	E	K	R	V			
CU‐12660 (H11N9)	H11N9	Ang Thong	A	A	T	L	D	A	V	G	K	R	V			
CU‐14442 (H11N9)	H11N9	Sukhothai	A	A	T	L	D	A	V	E	K	R	V			
CU‐16340 (H11N7)	H11N7	Kamphaengphet	A	A	T	L	D	A	V	E	K	R	V			

			NP													
Virus	Subtype	Location	16	61	100	109	214	283	293	305	313	357	372	422	442	455
Avian IAVs			G	V	I	R	R	L	R	R	F	Q	E	R	T	D
Human IAVs			D	I	L	V	K	P	K	K	Y	K	D	K	A	E
pdmH1N1 2009	H1N1	Ang Thong	G	I	I	V	R	L	R	K	V	K	E	R	T	D
CU‐12657 (H11N6)	H11N6	Ang Thong	G	V	I	R	R	L	R	R	F	Q	E	R	T	D
CU‐12677 (H11N6)	H11N6	Ang Thong	G	V	I	R	R	L	R	R	F	Q	E	R	T	D
CU‐12660 (H11N9)	H11N9	Ang Thong	G	V	I	R	R	L	R	R	F	Q	E	R	T	D
CU‐14442 (H11N9)	H11N9	Sukhothai	G	V	I	K	R	L	R	R	F	Q	E	R	T	D
CU‐16340 (H11N7)	H11N7	Kamphaengphet	G	V	I	R	R	L	K	H	L	Q	E	R	T	E

			M1			M2					NS1		NS2			
Virus	Subtype	Location	115	121	137	11	20	57	86	93	81	227	107			
Avian IAVs			V	T	T	T	S	Y	V	N	I	E	L			
Human IAVs			I	A	A	I	N	H	A	S	M	R	F			
pdmH1N1 2009	H1N1	Ang Thong	V	T	T	T	S	Y	V	N	I	del	L			
CU‐12657 (H11N6)	H11N6	Ang Thong	V	T	T	T	S	Y	V	N	I	E	L			
CU‐12677 (H11N6)	H11N6	Ang Thong	V	T	T	T	S	Y	V	N	I	E	L			
CU‐12660 (H11N9)	H11N9	Ang Thong	V	T	T	T	S	Y	V	N	I	E	L			
CU‐14442 (H11N9)	H11N9	Sukhothai	V	T	T	T	S	Y	V	N	I	E	L			
CU‐16340 (H11N7)	H11N7	Kamphaengphet	V	T	T	T	S	Y	V	N	I	E	L			

Abbreviation: IAV, influenza A virus.

## 4 DISCUSSION

FGD is one of the important reservoirs for influenza virus. FGDs can be infected with IAVs without clinical signs. Thus, FGDs can receive and/or spread IAVs to and/or from wild birds, domestic birds, domestic animals, and humans.^15^, ^17^ In Thailand, during HPAI‐H5N1 outbreaks, the FGD production system was considered to be an important potential risk pathway for HPAI‐H5N1 outbreaks.^14^ The HPAI‐H5N1 infected ducks showed no clinical signs with low mortality and morbidity. As FGDs can carry IAVs without clinical appearance and their nature of frequent movement among rice fields, these factors contributed to increasing risk of IAVs to widely spread in the country.^16^ Since 2008, the HPAI‐H5N1 outbreak has not been reported in Thailand; however, surveillance of IAV in FGDs has been routinely conducted to monitor the status of HPAI and LPAI viruses circulating in the country.

FGDs shared the habitats (rice fields) with wild birds and other domestic animals. Sharing habitat between wild birds and FGDs increased the risk of influenza virus transmission between both populations. For example, there was evidence that identical LPAI viruses could be isolated from both domestic ducks and wild birds in China. In Thailand, IAV subtypes H4N6, H4N9, H7N6, and H10N3 have been reported in Muscovy ducks housing in live bird markets.^34^, ^35^ Additionally, other LPAI viruses, IAV‐H12N1, were isolated from watercock and lesser‐whistling ducks.^35^ There are also several reports of IAVs circulating in ducks in Asia, for example, IAV‐H5N6 in Vietnam, IAV‐H7N4 in Cambodia, and IAV‐H5N3 in waterfowl and domestic ducks in China.^36^, ^37^ From the surveillance of IAVs in FGDs in Thailand during 2012–2015, we able to identify IAV‐H11 (n = 11). In 2012, the IAV‐H11N6 (n = 5) and IAV‐H11N9 (n = 2) could be isolated from FGD flocks in Ang Thong province. Then, in 2014, the IAV‐H11N9 (n = 1) was detected in FGD flock in Sukhothai province. In 2015, we identified IAV‐H11N7 (n = 3) in FGD flocks in Kamphaengphet province. Our results suggested that IAV‐H11 were constantly circulating in FGDs located in several provinces in Thailand during this period.

The phylogenetic analysis of Thai IAV‐H11 revealed two novel reassortant IAV‐H11 including rIAV‐H11N9 (CU12660) and rIAV‐H11N7 (CU16340). It has been known that the common lineages of eight gene segments of Thai IAVs is Eurasian lineage (EA); however, in this study, the novel reassortant IAV‐H11 contained gene segments originating from uncommon lineages. For example, the NP gene of IAV‐H11N9 (CU12660) and IAV‐H11N7 (CU16340) clusters with Asian group 1 (A1) and Asian group 2 (A2), respectively. Both groups were diverged from avian lineage before the separation of the North American (NA) and Eurasian lineage (EA). The Thai IAV‐H11N9 (CU12660) was clustered in Asian group 1 (A1) which includes IAV‐H3N8 from Vietnam and IAV‐H7N3 and IAV‐H7N7 from China,^38^ and Thai IAV‐H11N7 (CU16340) was clustered in Asian group 2 (A2), which included IAV‐H5N2 from China and Korea.^39^, ^40^ Interestingly, the M gene of IAV‐H11N7 (CU16340) was grouped into North American lineage (NA). The NS gene of IAV‐H11N9 (CU12660) was grouped into EA (allele B), whereas other Thai IAVs were grouped into EA (allele A) (EA). Because novel reassortment of Thai IAV‐H11 have been observed in this study suggesting that IAV‐H11 with internal gene segments originated from several sources are circulating in FGDs in Thailand, it should also be noted that novel reassortment of Thai IAV‐H11 was identified based on whole genome sequencing of five IAV‐H11. On the other hands, we could not conclude the reassortment of other IAV‐H11 with select gene segments (HA and NA) characterization.

Because most reported IAV‐H11 have been isolated from the Anseriformes order and few IAV‐H11N9 was detected in Charadriiformes order and rarely in Galliformes order.^41^ The FGDs are one of the reservoir species that are frequently infected with IAV‐H11. It has been reported that IAV‐H11 has zoonotic potential. There is evidence of IAV‐H11 exposure in humans, for example, seropositivity of H11 antibody among chicken growers, duck hunters, and wildlife professionals.^8^ In this study, Thai IAV‐H11 did not contain some virulence determinants, for example, PB2‐627 mutation (E627K), which relating to viral replication and more virulence of IAVs in mammals.^42^, ^43^ However, the Thai IAV‐H11N9 (CU12660) contained glycine (G) at PB2‐627, which is rarely reported in avian viruses and never been reported in mammalian viruses and need further investigation. For the analysis of genomic signatures, PA and NP gene segments of Thai IAV‐H11 contained identical amino acids in both avian and human viruses.

In summary, this study provided genetic information of Thai IAV‐H11 isolated from FGDs. The Thai IAV subtypes H11N6, H11N7, and H11N9 were characterized. Phylogenetic analysis showed that some IAV‐H11N9 and IAV‐H11N7 are novel reassortant viruses in Thailand. From amino acid analysis, the HA cleavage site and receptor biding sites of IAV‐H11 showed low pathogenic characteristics suggesting less potential to be zoonotic or virulence viruses. Novel reassortant IAV‐H11N9 and IAV‐H11N7 suggested that IAVs originated from several sources are circulating in FGDs in Thailand.

## AUTHOR CONTRIBUTIONS

**Supassama Chaiyawong:** Data curation; formal analysis; investigation; methodology. **Kamonpan Charoenkul:** Investigation; methodology; visualization. **Kitikhun Udom:** Investigation; methodology; visualization. **Ekkapat Chamsai:** Investigation; methodology; software. **Waleemas Jairak:** Formal analysis; investigation; methodology; validation; visualization. **Supanat Boonyapisitsopa:** Investigation; methodology. **Napawan Bunpapong:** Investigation; methodology. **Alongkorn Amonsin:** Conceptualization; formal analysis; supervision; validation.

### PEER REVIEW

The peer review history for this article is available at https://publons.com/publon/10.1111/irv.12960.

## Supporting information


**Table S1.** Detail of sample collection and IAV detection in this study.

**Table S2.** BLAST results of the nucleotide and amino acid identities of whole genome of Thai IAV‐H11 (H11‐N6, H11N7 and H11N9)

**Table S3.** BLAST results of the nucleotide and amino acid identities of HA and NA gene of Thai IAV‐H11 (H11‐N6, H11N7 and H11N9)

## Data Availability

The nucleotide sequence data that support the findings of this study are openly available in the GenBank database (https://www.ncbi.nlm.nih.gov/genbank/), under accession numbers MW857483‐857534.

## References

[1] Fouchier RA , Munster V , Wallensten A , et al. Characterization of a novel influenza A virus hemagglutinin subtype (H16) obtained from black‐headed gulls. J Virol. 2005;79(5):2814‐2822. doi:10.1128/JVI.79.5.2814‐2822.2005 15709000 10.1128/JVI.79.5.2814-2822.2005 PMC548452

[2] Steinhauer DA , Skehel JJ . Genetics of influenza viruses. Annu Rev Genet. 2002;36(1):305‐332. doi:10.1146/annurev.genet.36.052402.152757 12429695 10.1146/annurev.genet.36.052402.152757

[3] Tong S , Li Y , Rivailler P , et al. A distinct lineage of influenza A virus from bats. Proc Natl Acad Sci U S A. 2012;109(11):4269‐4274. doi:10.1073/pnas.1116200109 22371588 10.1073/pnas.1116200109 PMC3306675

[4] Tong S , Zhu X , Li Y , et al. New world bats harbor diverse influenza A viruses. PLoS Pathog. 2013;9(10):e1003657. doi:10.1371/journal.ppat.1003657 24130481 10.1371/journal.ppat.1003657 PMC3794996

[5] Hagag NM , Erfan AM , El‐Husseiny M , et al. Isolation of a novel reassortant highly pathogenic avian influenza (H5N2) virus in Egypt. Viruses. 2019;11(6). doi:10.3390/v11060565 10.3390/v11060565 PMC6631101 31216712

[6] Xu Y , Ramey AM , Bowman AS , et al. Low‐pathogenic influenza A viruses in North American diving ducks contribute to the emergence of a novel highly pathogenic influenza A(H7N8) virus. J Virol. 2017;91(9). doi:10.1128/JVI.02208‐16 10.1128/JVI.02208-16 PMC5391441 28202755

[7] Yang L , Xie J , Zhang Y , et al. Emergence of waterfowl‐originated gene cassettes in HPAI H7N9 viruses caused severe human infection in Fujian, China. Influenza Other Respi Viruses. 2019;13(5):496‐503. doi:10.1111/irv.12657 10.1111/irv.12657 PMC6692551 31187583

[8] Kayali G , Barbour E , Dbaibo G , et al. Evidence of infection with H4 and H11 avian influenza viruses among Lebanese chicken growers. PLoS ONE. 2011;6(10):e26818. doi:10.1371/journal.pone.0026818 22046370 10.1371/journal.pone.0026818 PMC3203926

[9] Pawar SD , Parkhi SS , Koratkar SS , Mishra AC . Receptor specificity and erythrocyte binding preferences of avian influenza viruses isolated from India. Virol J. 2012;9(1):251. doi:10.1186/1743‐422X‐9‐251 23110802 10.1186/1743-422X-9-251 PMC3502366

[10] Zhu H , Webby R , Lam TT , Smith DK , Peiris JS , Guan Y . History of swine influenza viruses in Asia. Curr Top Microbiol Immunol. 2013;370:57‐68. doi:10.1007/82_2011_179 21948002 10.1007/82_2011_179

[11] Chen C , Zhao G , Gu X , et al. Complete genomic sequence of a novel reassortant H11N3 influenza virus isolated from domestic ducks in Jiangsu, China. J Virol. 2012;86(21):11950‐11951. doi:10.1128/JVI.02167‐12 23043179 10.1128/JVI.02167-12 PMC3486340

[12] Wu H , Peng X , Peng X , Wu N . Molecular characterization of a reassortant H11N9 subtype avian influenza virus isolated from a domestic duck in Eastern China. Arch Virol. 2015;160(10):2595‐2601. doi:10.1007/s00705‐015‐2528‐6 26212362 10.1007/s00705-015-2528-6

[13] Zhang Y , Teng Q , Ren C , Li G , Li X , Li Z . Complete genome sequence of a novel reassortant H11N2 avian influenza virus isolated from a live poultry market in eastern China. J Virol. 2012;86(22):12443 doi:10.1128/JVI.02236‐12 23087108 10.1128/JVI.02236-12 PMC3486450

[14] Gilbert M , Chaitaweesub P , Parakamawongsa T , et al. Free‐grazing ducks and highly pathogenic avian influenza, Thailand. Emerg Infect Dis. 2006;12(2):227‐234. doi:10.3201/eid1202.050640 16494747 10.3201/eid1202.050640 PMC3373083

[15] Huang K , Bahl J , Fan XH , et al. Establishment of an H6N2 influenza virus lineage in domestic ducks in southern China. J Virol. 2010;84(14):6978‐6986. doi:10.1128/JVI.00256‐10 20463062 10.1128/JVI.00256-10 PMC2898240

[16] Hulse‐Post DJ , Sturm‐Ramirez KM , Humberd J , et al. Role of domestic ducks in the propagation and biological evolution of highly pathogenic H5N1 influenza viruses in Asia. Proc Natl Acad Sci U S A. 2005;102(30):10682‐10687. doi:10.1073/pnas.0504662102 16030144 10.1073/pnas.0504662102 PMC1180796

[17] Kim HR , Park CK , Lee YJ , et al. Low pathogenic H7 subtype avian influenza viruses isolated from domestic ducks in South Korea and the close association with isolates of wild birds. J Gen Virol. 2012;93(Pt 6):1278‐1287. doi:10.1099/vir.0.041269‐0 22422062 10.1099/vir.0.041269-0

[18] Songserm T , Jam‐on R , Sae‐Heng N , et al. Domestic ducks and H5N1 influenza epidemic, Thailand. Emerg Infect Dis. 2006;12(4):575‐581. doi:10.3201/eid1204.051614 16704804 10.3201/eid1204.051614 PMC3294714

[19] Li KS , Guan Y , Wang J , et al. Genesis of a highly pathogenic and potentially pandemic H5N1 influenza virus in eastern Asia. Nature. 2004;430(6996):209‐213. doi:10.1038/nature02746 15241415 10.1038/nature02746

[20] Song J , Feng H , Xu J , et al. The PA protein directly contributes to the virulence of H5N1 avian influenza viruses in domestic ducks. J Virol. 2011;85(5):2180‐2188. doi:10.1128/JVI.01975‐10 21177821 10.1128/JVI.01975-10 PMC3067757

[21] Waicharoen S , Thawatsupha P , Chittaganpitch M , Maneewong P , Thanadachakul T , Sawanpanyalert P . Influenza viruses circulating in Thailand in 2004 and 2005. Jpn J Infect Dis. 2008;61(4):321‐323. 18653981

[22] WHO . WHO Manual on Animal Influenza Diagnosis and Surveillance 2005. https://www.who.int/csr/resources/publications/influenza/whocdscsrncs20025rev.pdf

[23] Spackman E , Senne DA , Myers TJ , et al. Development of a real‐time reverse transcriptase PCR assay for type A influenza virus and the avian H5 and H7 hemagglutinin subtypes. J Clin Microbiol. 2002;40(9):3256‐3260. doi:10.1128/JCM.40.9.3256‐3260.2002 12202562 10.1128/JCM.40.9.3256-3260.2002 PMC130722

[24] Tsukamoto K , Ashizawa H , Nakanishi K , et al. Subtyping of avian influenza viruses H1 to H15 on the basis of hemagglutinin genes by PCR assay and molecular determination of pathogenic potential. J Clin Microbiol. 2008;46(9):3048‐3055. doi:10.1128/JCM.02386‐07 18596143 10.1128/JCM.02386-07 PMC2546769

[25] Tsukamoto K , Ashizawa T , Nakanishi K , et al. Use of reverse transcriptase PCR to subtype N1 to N9 neuraminidase genes of avian influenza viruses. J Clin Microbiol. 2009;47(7):2301‐2303. doi:10.1128/JCM.02366‐08 19403772 10.1128/JCM.02366-08 PMC2708491

[26] VanDalen KK , Anderson TD , Killian ML , Pedersen JC , Franklin AB , Piaggio AJ . Increased detection of influenza A H16 in the United States. Arch Virol. 2008;153(10):1981‐1983. doi:10.1007/s00705‐008‐0213‐8 18825483 10.1007/s00705-008-0213-8

[27] Hoffmann E , Stech J , Guan Y , Webster RG , Perez DR . Universal primer set for the full‐length amplification of all influenza A viruses. Arch Virol. 2001;146(12):2275‐2289. doi:10.1007/s007050170002 11811679 10.1007/s007050170002

[28] Ha Y , Stevens DJ , Skehel JJ , Wiley DC . X‐ray structure of the hemagglutinin of a potential H3 avian progenitor of the 1968 Hong Kong pandemic influenza virus. Virology. 2003;309(2):209‐218. doi:10.1016/S0042‐6822(03)00068‐0 12758169 10.1016/s0042-6822(03)00068-0

[29] Matrosovich M , Tuzikov A , Bovin N , et al. Early alterations of the receptor‐binding properties of H1, H2, and H3 avian influenza virus hemagglutinins after their introduction into mammals. J Virol. 2000;74(18):8502‐8512. doi:10.1128/JVI.74.18.8502‐8512.2000 10954551 10.1128/jvi.74.18.8502-8512.2000 PMC116362

[30] Gaymard A , Charles‐Dufant A , Sabatier M , et al. Impact on antiviral resistance of E119V, I222L and R292K substitutions in influenza A viruses bearing a group 2 neuraminidase (N2, N3, N6, N7 and N9). J Antimicrob Chemother. 2016;71(11):3036‐3045. doi:10.1093/jac/dkw275 27432605 10.1093/jac/dkw275

[31] Song MS , Marathe BM , Kumar G , et al. Unique determinants of neuraminidase inhibitor resistance among N3, N7, and N9 avian influenza viruses. J Virol. 2015;89(21):10891‐10900. doi:10.1128/JVI.01514‐15 26292325 10.1128/JVI.01514-15 PMC4621141

[32] Li J , Ishaq M , Prudence M , et al. Single mutation at the amino acid position 627 of PB2 that leads to increased virulence of an H5N1 avian influenza virus during adaptation in mice can be compensated by multiple mutations at other sites of PB2. Virus Res. 2009;144(1‐2):123‐129. doi:10.1016/j.virusres.2009.04.008 19393699 10.1016/j.virusres.2009.04.008

[33] Schat KA , Bingham J , Butler JM , et al. Role of position 627 of PB2 and the multibasic cleavage site of the hemagglutinin in the virulence of H5N1 avian influenza virus in chickens and ducks. PLoS ONE. 2012;7(2):e30960. doi:10.1371/journal.pone.0030960 22363523 10.1371/journal.pone.0030960 PMC3283584

[34] Jairak W , Boonyapisitsopa S , Chaiyawong S , et al. Genetic characterization of influenza A (H7N6) virus isolated from a live‐bird market in Thailand. Arch Virol. 2016;161(5):1315‐1322. doi:10.1007/s00705‐016‐2759‐1 26795160 10.1007/s00705-016-2759-1

[35] Wongphatcharachai M , Wisedchanwet T , Lapkuntod J , Nonthabenjawan N , Jairak W , Amonsin A . Genetic characterization of influenza A virus subtype H12N1 isolated from a watercock and lesser whistling ducks in Thailand. Arch Virol. 2012;157(6):1123‐1130. doi:10.1007/s00705‐012‐1260‐8 22367500 10.1007/s00705-012-1260-8

[36] Vijaykrishna D , Deng YM , Grau ML , et al. Emergence of Influenza A(H7N4) Virus, Cambodia. Emerg Infect Dis. 2019;25(10):1988‐1991. doi:10.3201/eid2510.190506 31310233 10.3201/eid2510.190506 PMC6759271

[37] Tsunekuni R , Sudo K , Nguyen PT , et al. Isolation of highly pathogenic H5N6 avian influenza virus in Southern Vietnam with genetic similarity to those infecting humans in China. Transbound Emerg Dis. 2019;66(6):2209‐2217. doi:10.1111/tbed.13294 31309743 10.1111/tbed.13294

[38] Cui P , Deng G , Shi J , et al. New influenza A(H7N7) viruses detected in live poultry markets in China. Virology. 2016;499:165‐169. doi:10.1016/j.virol.2016.06.015 27661735 10.1016/j.virol.2016.06.015

[39] Mi Z , Liu W , Fan H , et al. Complete genome sequence of avian influenza virus A/chicken/Jiangsu/1001/2013(H5N2), demonstrating continuous reassortance of H5N2 in China. Genome Announc. 2013;1(4):e00469‐13. doi:10.1128/genomeA.00469‐13 23868124 10.1128/genomeA.00469-13 PMC3715666

[40] Kim HR , Park CK , Oem JK , et al. Characterization of H5N2 influenza viruses isolated in South Korea and their influence on the emergence of a novel H9N2 influenza virus. J Gen Virol. 2010;91(Pt 8):1978‐1983. doi:10.1099/vir.0.021238‐0 20392898 10.1099/vir.0.021238-0

[41] Li J , Cardona CJ , Xing Z , Woolcock PR . Genetic and phenotypic characterization of a low‐pathogenicity avian influenza H11N9 virus. Arch Virol. 2008;153(10):1899‐1908. doi:10.1007/s00705‐008‐0217‐4 18825481 10.1007/s00705-008-0217-4

[42] Long JS , Howard WA , Nunez A , et al. The effect of the PB2 mutation 627K on highly pathogenic H5N1 avian influenza virus is dependent on the virus lineage. J Virol. 2013;87(18):9983‐9996. doi:10.1128/JVI.01399‐13 23843645 10.1128/JVI.01399-13 PMC3753988

[43] Hatta M , Gao P , Halfmann P , Kawaoka Y . Molecular basis for high virulence of Hong Kong H5N1 influenza A viruses. Science. 2001;293(5536):1840‐1842. doi:10.1126/science.1062882 11546875 10.1126/science.1062882

