# Supplementary material for: Genetic characterization of influenza A virus subtypes H11N6, H11N7, and H11N9 isolated from free‐grazing ducks, Thailand
Source: Influenza Other Respir Viruses. 2022 Jan 10;16(4):726–39. doi: 10.1111/irv.12960 (PMC9178063; doi:10.1111/irv.12960)
Supplement: Supplementary file 1 — Table S1. Detail of sample collection and IAV detection in this study. [file IRV-16-726-s001.docx]

**Supplement Material**

**Genetic characterization of influenza A virus subtypes H11N6, H11N7 and H11N9**

**isolated from free-grazing ducks, Thailand**

Supassama Chaiyawong^1,2^, Kamonpan Charoenkul^1,2^, Kitikhun Udom^1,2^, Ekkapat Chamsai^1,2^

Waleemas Jairak^1,2^, Supanat Boonyapisitsopa^2,3^, Napawan Bunpapong^2,3^,

Alongkorn Amonsin^1,2*^

^1^Department of Veterinary Public Health, Faculty of Veterinary Science, Chulalongkorn University, Bangkok, 10330, Thailand

^2^Emerging and Re-emerging Infectious Diseases in Animals, Center of Excellence, and One Health Research Cluster, Faculty of Veterinary Science, Chulalongkorn University, Bangkok, 10330, Thailand

^3^Veterinary Diagnostic Laboratory, Faculty of Veterinary Science, Chulalongkorn University, Bangkok, 10330, Thailand

**Supplement Table**

**Supplement Table 1.** Detail of sample collection and IAV detection in this study.

**Supplement Table 2.** BLAST results of the nucleotide and amino acid identities of whole genome of Thai IAV-H11 (H11-N6, H11N7 and H11N9)

**Supplement Table 3.** BLAST results of the nucleotide and amino acid identities of HA and NA gene of Thai IAV-H11 (H11-N6, H11N7 and H11N9)

**Supplement Table 1.** Detail of sample collection and IAV detection in this study.

| Date | # of FGD flocks | # of samples |  | # positive  RT-PCR | Subtype identification |
| --- | --- | --- | --- | --- | --- |
|  |  | Oropharyngeal swab | Cloacal swab |  |  |
|  |  |  |  |  |  |
| 2012-2013 | 6 | 290 | 290 | 13 (2.24%) | H11N6 (n=5), H11N9 (n=2), H4N6 (n=4), Unknown* (n=2) |
| 2013-2014 | 7 | 330 | 330 | 18 (2.73%) | H4N6 (n=6), H4N9 (n=1), H3N8 (n=7), Unknown* (n=4) |
| 2014-2015 | 5 | 160 | 160 | 4 (1.25%) | H11N9 (n=1)  H11N7 (n=3) |
|  |  |  |  |  |  |
|  |  | 780 | 780 | 35 (2.24%) |  |
|  |  |  |  |  |  |

Unknown*, unknown subtype due to low virus titer

**Supplement Table 2.** BLAST results of the nucleotide and amino acid identities of whole genome of Thai IAV-H11 (H11-N6, H11N7 and H11N9)

| Virus | Gene | Closet reference virus | % nucleotide identity |
| --- | --- | --- | --- |
| CU-12657 (H11N6) | | | |
|  | PB2 | A/ruddy shelduck/Mongolia/974/2010 (H10N7) | 98.19% |
|  | PB1 | A/duck/Vietnam/LBM48/2011(H3N2) | 99.03% |
|  | PA | A/duck/Vietnam/LBM48/2011(H3N2) | 98.93% |
|  | HA11 | A/duck/Thailand/CU5388/2009 (H11N3) | 95.65% |
|  | NP | A/muscovy duck/Vietnam/LBM115/2012 (H3N2) | 99.01% |
|  | NA6 | A/duck/Thailand/CU-12679C/2012 (H4N6) | 98.93% |
|  | M | A/duck/Jiangxi/3008/2009 (mixed) | 98.92% |
|  | NS | A/wild duck/Korea/SH5-26/2008 (H4N6) | 99.10% |
| CU-12677 (H11N6) | | | |
|  | PB2 | A/ruddy shelduck/Mongolia/974/2010(H10N7) | 98.25% |
|  | PB1 | A/duck/Vietnam/LBM48/2011(H3N2) | 99.12% |
|  | PA | A/duck/Vietnam/LBM48/2011(H3N2) | 98.88% |
|  | HA11 | A/duck/Thailand/CU5388/2009(H11N3) | 99.27% |
|  | NP | A/aquatic bird/Korea/w344/2008(H5N2) | 99.13% |
|  | NA6 | A/duck/Thailand/CU-12679C/2012 (H4N6) | 98.87% |
|  | M | A/duck/Jiangxi/3008/2009(mixed) | 99.02% |
|  | NS | A/wild duck/Korea/SH5-26/2008(H4N6) | 99.10% |
| CU-12660 (H11N9) | | | |
|  | PB2 | A/duck/Mongolia/121/2011(H10N7) | 98.38% |
|  | PB1 | A/duck/Thailand/CU-11869C/2011(H1N9) | 98.84% |
|  | PA | A/duck/Vietnam/G17-1/2011 (H11N9) | 98.93% |
|  | HA11 | A/duck/Thailand/CU5388/2009 (H11N3) | 95.47% |
|  | NP | A/duck/Wenzhou/771/2013 (H7N3) | 97.24% |
|  | NA9 | A/duck/Jiangxi/3096/2009 (H7N9) | 97.31% |
|  | M | A/duck/Vietnam/G17-1/2011 (H11N9) | 98.71% |
|  | NS | A/duck/Vietnam/OIE-0483/2012 (H10N7) | 98.45% |
| CU-14442 (H11N9) | | | |
|  | PB2 | A/duck/Jiangxi/33641/2013 (H10N3) | 99.52% |
|  | PB1 | A/Anseriformes/Anhui/L25/2014 (H1N1) | 99.47% |
|  | PA | A/common teal/Nanji/NJ-280/2013 (H6N1) | 99.21% |
|  | HA11 | A/duck/Jiangxi/22537/2012 (H11N9) | 98.88% |
|  | NP | A/duck/Jiangxi/33775/2013 (H10N3) | 99.32% |
|  | NA9 | A/mallard/Sweden/101011/2009 (H11N9) | 98.75% |
|  | M | A/chicken/Jilin/SD001/2014 (H9N2) | 99.90% |
|  | NS | A/chicken/Jiangxi/C25/2014 (H7N7) | 99.28% |
| CU-16340 (H11N7) | | | |
|  | PB2 | A/duck/Mongolia/123/2014 (H10N8) | 99.52% |
|  | PB1 | A/duck/Hunan/S4120/2011 (H5N2) | 98.42% |
|  | PA | A/duck/Hunan/S4101/2011 (H5N2) | 98.40% |
|  | HA11 | A/duck/Jiangxi/22537/2012 (H11N9) | 98.78% |
|  | NP | A/duck/Hunan/S4120/2011 (H5N2) | 99.12% |
|  | NA7 | A/duck/Mongolia/709/2015 (H10N7) | 99.20% |
|  | M | A/mallard/Hokkaido/24/2009 (H5N1) | 98.66% |
|  | NS | A/duck/Hokkaido/W19/2013 (H7N2) | 99.28% |

**Supplement Table 3.** BLAST results of the nucleotide and amino acid identities of HA and NA gene of Thai IAV-H11 (H11-N6, H11N7 and H11N9)

| Virus | Gene | Closet reference virus | % nucleotide identity |
| --- | --- | --- | --- |
| CU-12657 (H11N6) | | | |
|  | HA11 | A/duck/Thailand/CU5388/2009 (H11N3) | 95.65% |
|  | NA6 | A/duck/Thailand/CU-12679C/2012 (H4N6) | 98.93% |
| CU-12658 (H11N6) | | | |
|  | HA11 | A/duck/Thailand/CU5388/2009 (H11N3) | 95.59% |
|  | NA6 | A/duck/Thailand/CU-11655T/2011(H4N6) | 97.92% |
| CU-12661 (H11N6) | | | |
|  | HA11 | A/duck/Thailand/CU5388/2009 (H11N3) | 95.46% |
|  | NA6 | A/duck/Thailand/CU-12679C/2012 (H4N6) | 98.94% |
| CU-12677 (H11N6) | | | |
|  | HA11 | A/duck/Thailand/CU5388/2009(H11N3) | 99.27% |
|  | NA6 | A/duck/Thailand/CU-12679C/2012 (H4N6) | 98.87% |
| CU-12678 (H11N6) | | | |
|  | HA11 | A/duck/Thailand/CU5388/2009 (H11N3) | 95.48% |
|  | NA6 | A/duck/Thailand/CU-12679C/2012(H4N6) | 98.66% |
| CU-12660 (H11N9) | | | |
|  | HA11 | A/duck/Thailand/CU5388/2009 (H11N3) | 95.47% |
|  | NA9 | A/duck/Jiangxi/3096/2009 (H7N9) | 97.31% |
| CU-12662 (H11N9) | | | |
|  | HA11 | A/duck/Thailand/CU5388/2009 (H11N3) | 95.44% |
|  | NA9 | A/duck/Jiangxi/3096/2009 (H7N9) | 97.04% |
| CU-14442 (H11N9) | | | |
|  | HA11 | A/duck/Jiangxi/22537/2012 (H11N9) | 98.88% |
|  | NA9 | A/mallard/Sweden/101011/2009 (H11N9) | 98.75% |
| CU-16340 (H11N7) | | | |
|  | HA11 | A/duck/Jiangxi/22537/2012 (H11N9) | 98.78% |
|  | NA7 | A/duck/Mongolia/709/2015 (H10N7) | 99.20% |
| CU-16345 (H11N7) | | | |
|  | HA11 | A/duck/Jiangxi/22537/2012 (H11N9) | 98.86% |
|  | NA7 | A/duck/Mongolia/709/2015 (H10N7) | 99.27% |
| CU-16347 (H11N7) | | | |
|  | HA11 | A/duck/Jiangxi/22537/2012 (H11N9) | 98.84% |
|  | NA7 | A/duck/Mongolia/709/2015 (H10N7) | 99.27% |
